# Enhanced Bioactivities of Fermented *Rehmannia glutinosa* via Catalpol-Mediated GLP-1R Signaling

**DOI:** 10.3390/cimb48060559

**Published:** 2026-05-26

**Authors:** Eun-Ji You, Boyong Kim

**Affiliations:** 1 Sun Dental Hospital, 645 Daejong-ro, Jung-gu, Daejeon 34925, Republic of Korea; youeunji90@nate.com; 2 Association for Cannabis Regulatory Science, 102, 98-12 Suho-ro, Pungcheon-myeon, Andong 36729, Republic of Korea; 3 EVERBIO, 131, Jukhyeon-gil, Gwanghyewon-myeon, Jincheon 27809, Republic of Korea

**Keywords:** *Rehmannia glutinosa*, fermentation, catalpol, GLP-1 receptor, MUC2, SGLT1, GLUT2, intestinal glucose absorption

## Abstract

Fermentation is widely used to enhance the bioactivity of herbal phytochemicals through microbial bioconversion. *Rehmannia glutinosa* contains catalpol, an iridoid glycoside with metabolic and immunomodulatory potential; however, its efficacy in the unfermented form is limited. This study investigated whether fermentation enhances catalpol production and improves metabolic and immune-regulating functions via glucagon-like peptide-1 receptor (GLP-1R) signaling. *Rehmannia glutinosa* extract was fermented under optimized conditions, and catalpol and iridoid precursor levels were quantified to assess bioconversion efficiency. Biological effects were evaluated in intestinal epithelial cells, macrophages, and an Artemia model, focusing on glucose transport, GLP-1 secretion, dipeptidyl peptidase-4 (DPP-4) expression, mucosal defense, and GLP-1R/protein kinase A/cAMP response element-binding protein (PKA/CREB) signaling. Fermentation significantly increased catalpol content while reducing iridoid precursors. The fermented extract suppressed intestinal glucose absorption by downregulating sodium–glucose cotransporter 1 (SGLT1) and glucose transporter 2 (GLUT2). It also enhanced GLP-1 secretion and reduced DPP-4 expression, leading to activation of GLP-1R/PKA/CREB signaling. This activation increased mucin 2 (MUC2) expression and promoted anti-inflammatory.

## 1. Introduction

Fermentation is increasingly recognized as an effective strategy to enhance the bioavailability and biological activity of herbal phytochemicals through microbial bioconversion processes [1,2]. In particular, fermentation facilitates the conversion of precursor compounds into more bioactive metabolites, thereby amplifying their pharmacological effects [1].

*Rehmannia glutinosa* is a widely used medicinal herb with established metabolic and immunomodulatory properties, largely attributed to its iridoid glycosides such as catalpol [3]. Catalpol has been reported to exert hypoglycemic, anti-inflammatory, and mucosal protective activities through regulation of glucose homeostasis, oxidative stress responses, and cytokine signaling [4,5,6]. However, the intrinsic bioactivity of unfermented extracts is often limited by insufficient conversion efficiency and bioavailability of active metabolites. Catalpol is synthesized through a conserved iridoid glycoside biosynthetic pathway, in which loganic acid, loganin, and aucubin act as upstream precursors [7]. The conversion of these precursors into catalpol requires sequential deglycosylation, ester cleavage, and oxidation reactions. Microbial fermentation is known to accelerate such biotransformation processes. In particular, lactic acid bacteria, including *Lactobacillus plantarum*, produce carbohydrate-active enzymes such as β-glucosidase, esterase, and oxidoreductase, which facilitate glycoside hydrolysis and structural remodeling of iridoid intermediates, thereby enhancing the conversion efficiency of iridoid precursors into catalpol [8,9].

Recent studies have demonstrated that fermentation by *Lactobacillus* species significantly increases catalpol availability through enzyme-mediated hydrolysis of glycosidic bonds, suggesting that fermentation-driven bioconversion is a key determinant of catalpol enrichment [10,11]. Several previous studies have also reported the successful application of *Lactobacillus*-mediated fermentation in herbal extracts to enhance the conversion and bioavailability of glycoside-derived phytochemicals [8,9]. Fermented *Rehmannia glutinosa* extracts (FRGE) have been reported to exhibit enhanced antioxidant and immunomodulatory activities compared with non-fermented extracts [12,13]. However, the functional consequences of fermentation-induced catalpol enrichment remain poorly defined. In particular, it remains unclear whether FRGE directly modulates key intestinal glucose transporters such as sodium–glucose cotransporter 1 (SGLT1) and glucose transporter 2 (GLUT2), which are essential determinants of luminal glucose absorption [14].

Although catalpol has been reported to influence incretin pathways, including stimulation of glucagon-like peptide-1 (GLP-1) secretion and activation of GLP-1 receptor signaling, its role in coordinating intestinal glucose transport and mucosal immune responses remains insufficiently understood [15,16,17]. Furthermore, whether fermentation-induced catalpol enrichment contributes to GLP-1 activity by altering the balance between GLP-1 production and degradation, particularly via dipeptidyl peptidase-4 (DPP-4), has not been elucidated. GLP-1 signaling plays a pivotal role not only in metabolic regulation but also in immune modulation. Activation of GLP-1 receptor (GLP-1R) has been shown to promote anti-inflammatory responses, including increased secretion of cytokines such as interleukin (IL)-4, IL-10, and IL-22, through downstream signaling pathways involving protein kinase A (PKA) and cAMP response element-binding protein (CREB) [16,18]. Moreover, GLP-1R activation contributes to the maintenance of intestinal barrier integrity by enhancing mucin production, particularly mucin 2 (MUC2), which plays a critical role in mucosal defense [19,20].

Despite these insights, the role of fermentation-enhanced catalpol in modulating GLP-1R-mediated signaling pathways, particularly in the context of intestinal glucose transport and mucosal immune responses, remains to be fully elucidated. Importantly, the causal involvement of GLP-1 signaling in mediating the biological effects of fermented *Rehmannia glutinosa* extract has not been experimentally validated.

This study aimed to investigate whether fermentation enhances the biological activity of *Rehmannia glutinosa* extract through catalpol enrichment and to determine whether these effects are mediated via GLP-1R-dependent signaling pathways. Specifically, we evaluated intestinal glucose transport, incretin regulation, mucosal defense, and macrophage-mediated immune responses. Furthermore, we systematically validated the mechanistic role of GLP-1 signaling in mediating these effects. Unlike previous studies primarily focused on antioxidant or general anti-inflammatory activities of fermented *Rehmannia glutinosa*, the present study demonstrates that fermentation-enhanced catalpol enrichment regulates intestinal glucose transport through suppression of SGLT1 and GLUT2 while simultaneously activating GLP-1R-dependent mucosal immune signaling. Furthermore, the mechanistic involvement of catalpol-mediated GLP-1R signaling was experimentally validated using a GLP-1R antagonist.

## 2. Materials and Methods

### 2.1. Extraction, Fermentation, and Catalpol Analysis

*R. glutinosa* roots were extracted using 50% ethanol (Sigma-Aldrich, St. Louis, MO, USA) at a solid-to-solvent ratio of 1:10 (*w*/*v*) for 24 h at 25 °C under dark conditions. Following filtration and concentration, the extraction yield was approximately 18.6% (*w*/*w*). For in vitro assays, FRGE and RGE were used at a final concentration of 100 μg/mL unless otherwise indicated. The extract was sonicated at 25 °C, 60 kHz for 60 min (SD-D200H; Sungdong, Seoul, Republic of Korea) and subsequently subjected to fermentation. Fermentation was performed using *Lactobacillus plantarum* obtained from the Korean Culture Center of Microorganisms (Seoul, Republic of Korea). Anaerobic fermentation was conducted at 35 °C for 72 h. Two fermentation conditions were applied: general fermentation (GF) and specific fermentation (SF). The GF medium contained 0.5% glucose and 0.1% yeast extract (Thermo Fisher Scientific, Waltham, MA, USA). The SF condition was optimized based on preliminary experiments and previous reports on *Lactobacillus*-mediated iridoid bioconversion to enhance precursor-to-catalpol conversion, using pH 5.2 ± 0.1, an inoculum density of 2% (*v*/*v*), reduced nutrient supplementation (0.1% glucose and 0.05% yeast extract), and a shortened fermentation duration of 48 h. Catalpol and iridoid precursors were quantified using an Agilent 1260 Infinity II HPLC system (Agilent Technologies, Santa Clara, CA, USA) equipped with a ZORBAX Eclipse Plus C18 column (Agilent Technologies, Santa Clara, CA, USA). Detection was performed at 210 nm, and quantification was based on calibration curves using authentic standards.

### 2.2. Cell Culture and Treatment

Caco-2 cells and NCI-H716 intestinal L cells (ATCC, Manassas, VA, USA) were cultured under standard conditions. Macrophages (KG-1, ATCC, Manassas, VA, USA) were cultured separately for immune response analysis [17]. Cells were exposed to multiple experimental conditions depending on the assay, including control (Con), RGE, FRGE, high glucose (HG; 25 mM), FRGE + HG, catalpol + HG, Exendin (9–39) + HG, FRGE + HG + Exendin (9–39), and catalpol + HG + Exendin (9–39) for 24 h. For GLP-1 secretion assays, cells were treated for up to 10 days to evaluate chronic metabolic responses. Cytotoxicity was assessed using a propidium iodide (PI)–Annexin V apoptosis assay (Thermo Fisher Scientific, Waltham, MA, USA), and non-cytotoxic concentrations were used for all experiments.

### 2.3. Glucose Uptake Assay

Glucose uptake in Caco-2 cells was evaluated using a fluorescent glucose analog (Cayman Chemical, Ann Arbor, MI, USA). Cells were incubated with the fluorescent glucose analog for 30 min at 37 °C prior to analysis [17]. After treatment, cells were washed with PBS (Thermo Fisher Scientific, Waltham, MA, USA) and incubated in glucose-free DMEM (Thermo Fisher Scientific, Waltham, MA, USA). Glucose uptake was then analyzed using a BD FACSCalibur flow cytometer (BD Biosciences, Franklin Lakes, NJ, USA). Fluorescence intensity was quantified to determine relative glucose uptake.

### 2.4. In Vivo Glucose Absorption (Artemia Model)

*Artemia franciscana* nauplii were cultured in artificial seawater at 30 °C (pH 8.0) and exposed to experimental conditions for 24 h. Subsequently, nauplii were incubated with FITC-labeled glucose (10 mg/mL) for 36 h, a concentration selected based on preliminary optimization for visualization sensitivity [17]. Fluorescence distribution was analyzed using an Eclipse Ts-2 fluorescence microscope (Nikon, Corporation, Tokyo, Japan), and glucose uptake was quantified by counting FITC-positive regions.

### 2.5. Enzyme-Linked Immunosorbent Assay (ELISA)

GLP-1 secretion and DPP-4 expression were measured using commercial ELISA kits (Thermo Fisher Scientific, Waltham, MA, USA) according to the manufacturer’s instructions. Cells were exposed to Con, HG, FRGE, and FRGE + HG conditions. For immune analysis, conditioned media from treated Caco-2 cells were applied to macrophages, and cytokine secretion (IL-4, IL-10, IL-22) was quantified using ELISA. Absorbance was measured using a microplate reader (AMR-100, Allsheng, Hangzhou, China).

### 2.6. Flow Cytometry

Cells were stained with primary antibodies (1:200 dilution) for 30 min at 4 °C against SGLT1, GLUT2, GLP-1R, phospho-PKA, and CREB (Thermo Fisher Scientific, Waltham, MA, USA). Data were acquired using a BD FACSCalibur flow cytometer and analyzed using FlowJo software (v10.10.1).

### 2.7. GLP-1R Inhibition Assay

To validate the involvement of catalpol-mediated GLP-1R signaling in mucosal defense, additional experiments were performed using catalpol and a GLP-1R antagonist. Caco-2 cells were treated under the following conditions for 24 h: control (Con), high glucose (HG; 25 mM), catalpol + HG, FRGE + HG, Exendin (9–39) + HG, FRGE + HG + Exendin (9–39), and catalpol + HG + Exendin (9–39). Catalpol (>98% purity; Chengdu Must Bio-Technology Co., Ltd., Chengdu, China) was applied at a concentration of 10 µM. The GLP-1R antagonist Exendin (9–39) (Sigma-Aldrich, St. Louis, MO, USA) was used at 100 nM and added 1 h prior to catalpol or FRGE treatment. Catalpol was applied for 24 h following Exendin (9–39) pretreatment. After treatment, MUC2 levels were quantified using a commercial ELISA kit (Thermo Fisher Scientific) according to the manufacturer’s instructions. Absorbance was measured using a microplate reader (AMR-100, Allsheng, Hangzhou, China), and relative MUC2 expression levels were calculated based on standard curves.

### 2.8. Statistical Analysis

All experimental data were analyzed using one-way analysis of variance (ANOVA) followed by Scheffé’s post hoc test (GraphPad Prism version 8.0; GraphPad Software, San Diego, CA, USA). Data are presented as mean ± SEM, and statistical significance was defined as *p* < 0.05. All experiments were performed with at least three biological replicates (n = 3 independent biological replicates). Fold-change values described in the Results section were calculated based on mean values obtained from independent biological replicates and were considered significant only when supported by statistical analysis (*p* < 0.05).

## 3. Results

### 3.1. Enhanced Bioactive Compounds and Glucose-Regulating Effects of FRGE

**Figure 1:**
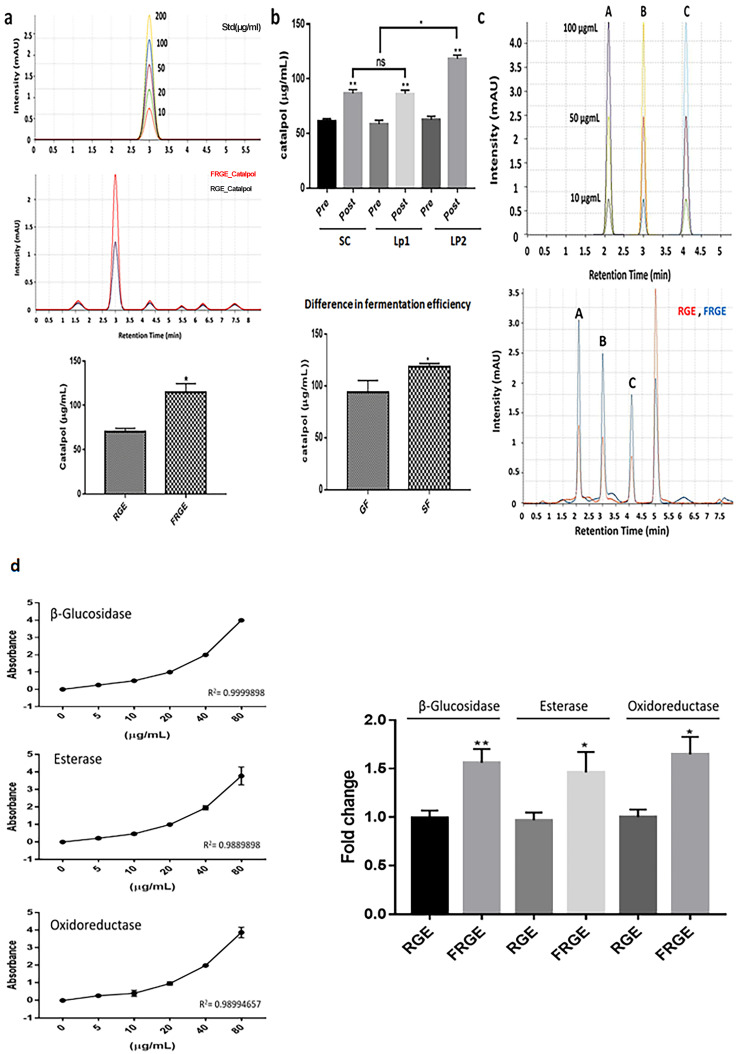
Quantitative analysis of catalpol under different fermentation conditions of *Rehmannia glutinosa* extract. (**a**) Analytical results for Catalpol in FRGE and RGE using HPLC. (**b**) Upper panel: HPLC-based quantification of catalpol levels before and after fermentation using various microbial strains (SC: *Saccharomyces cerevisiae*; LP1: *Lactobacillus plantarum* ATCC 8014, *Lactobacillus plantarum* KCCM 43246). Lower panel: comparative analysis of catalpol levels under general (GF) and specific fermentation (SF) conditions. (**c**) Quantitative analysis of the three major precursors (A: Loganic acid, B: Loganin, C: Aucubin) involved in catalpol biosynthesis in RGE and FRGE. (**d**) Assessment of the synthesis levels of specific microbial enzymes following exposure to the extract. FRGE, fermented *Rehmannia glutinosa* root extract; RGE, *Rehmannia glutinosa* root extract; Std, standard; ns, not significant (* *p* < 0.05, ** *p* < 0.01).

A comparison of the catalpol content between FRGE and RGE revealed concentrations of approximately 62.4 µg/mL in RGE and 116.7 µg/mL in FRGE, corresponding to an approximately 1.87 times increase (Figure 1a). This fermentation-induced enhancement supports the notion that catalpol levels are markedly elevated following microbial processing. In panel B, catalpol content significantly increased across all fermentation conditions. *Saccharomyces cerevisiae* (SC), *Lactobacillus plantarum* ATCC 8014 (LP1), and *L. plantarum* KCCM 43246 (LP2) each exhibited higher catalpol concentrations post-fermentation than their respective pre-fermentation levels, with LP2 showing the greatest increase (Table 1). Furthermore, when LP2 was fermented under two distinct processing protocols—general fermentation (GF) and specific fermentation (SF)—the SF protocol yielded substantially higher catalpol levels than GF (Figure 1b). Because the same microbial strain was used for both protocols, this difference suggests that optimized fermentation parameters, rather than strain variability, account for the enhanced catalpol production.

Analysis of iridoid precursors showed marked reductions after fermentation, with loganic acid, loganin, and aucubin decreasing by approximately 42%, 38%, and 47%, respectively (Figure 1c). These decreases were accompanied by a 1.5 to 2 times increase in catalpol content, consistent with fermentation-driven metabolic conversion of precursors into catalpol. In parallel, the activities of β-glucosidase, esterase, and oxidoreductase increased by 1.7, 1.5, and 1.6 times, respectively, in fermented samples, supporting an enzyme-mediated mechanism that facilitates iridoid remodeling and catalpol enhancement (Figure 1d).

**Figure 2:**
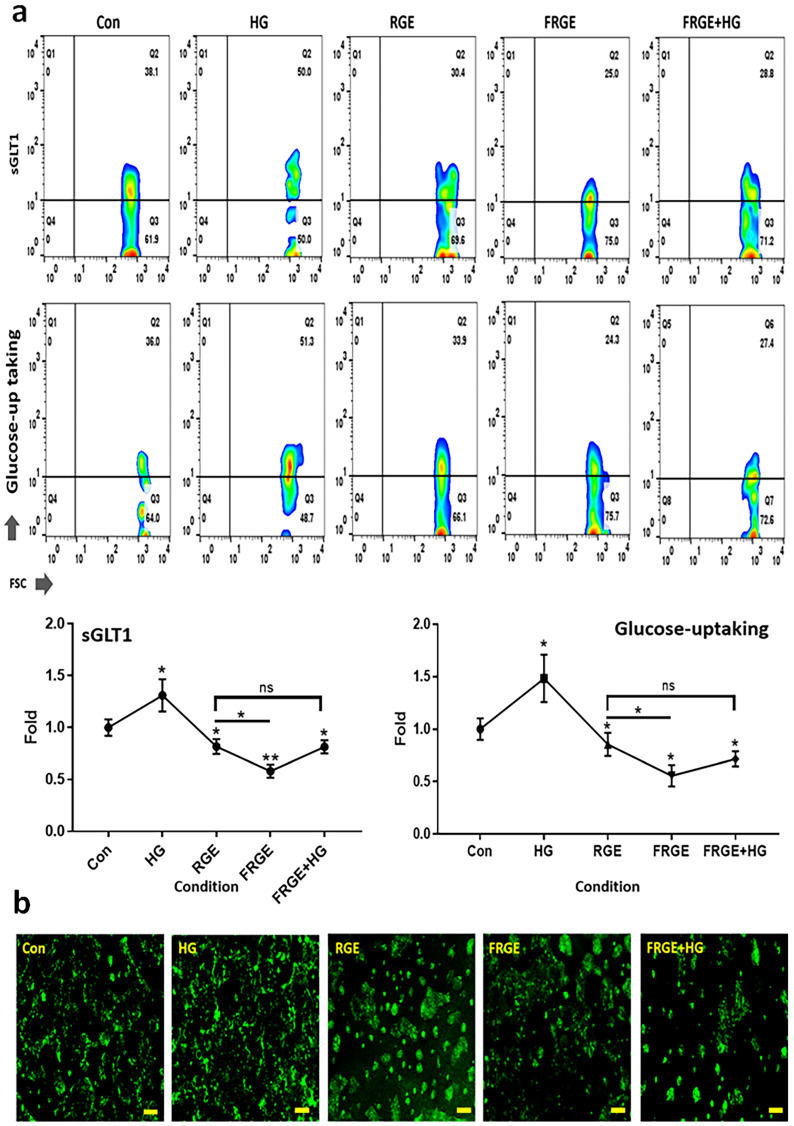
Modulation of glucose absorption by FRGE in intestinal cells. (**a**) Quantitative analysis of SGLT1 expression and fluorescent glucose uptake in Caco-2 intestinal epithelial cells under different experimental conditions. (**b**) Representative fluorescence microscopy images showing SGLT1-positive intestinal epithelial cells under control (Con), high glucose (HG), RGE, FRGE, and FRGE + HG conditions. Experimental group labels were added directly to each panel for clarity. Images were captured under identical exposure settings. Con, control; HG, high glucose exposure; FRGE, fermented *Rehmannia glutinosa* root extract; RGE, *Rehmannia glutinosa* root extract; FRGE + HG, mixture with FRGE and HG; ns, not significant (* *p* < 0.05, ** *p* < 0.01) (Scale bars = 20 μm).

The inhibitory effect of FRGE on SGLT1 expression was evaluated. SGLT1 expression decreased by approximately 30% compared with RGE exposure (Figure 2a). Notably, under HG conditions, SGLT1 expression in the FRGE-treated group remained suppressed by approximately 20% relative to the control group and showed an approximately 1.6 times decrease compared with the other HG-exposed groups (Figure 2a). Immunocytochemical analysis revealed a pattern consistent with the flow cytometry findings (Figure 2b).

**Figure 3:**
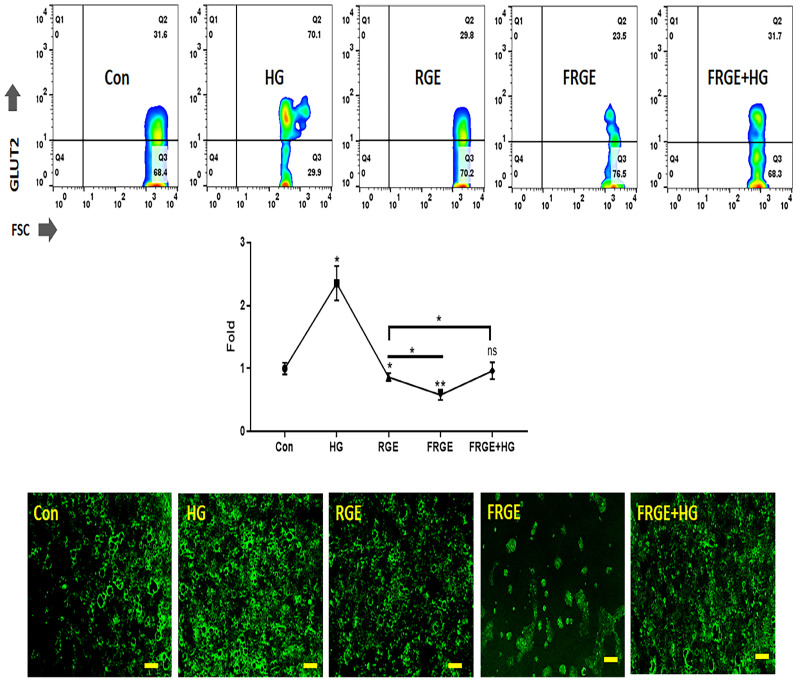
FRGE suppresses GLUT2 expression in intestinal epithelial cells. Quantitative analysis of GLUT2 expression in intestinal epithelial cells under different treatment conditions. Representative fluorescence microscopy images showing FITC-GLUT2-positive cells under Con, HG, RGE, FRGE, and FRGE + HG conditions. Experimental group labels were added directly to each panel to improve interpretability. Images were acquired using identical exposure conditions. Con, control; HG, high glucose exposure; FRGE, fermented *Rehmannia glutinosa* root extract; RGE, *Rehmannia glutinosa* root extract; FRGE + HG, mixture with FRGE and HG, ns; not significant (* *p* < 0.05, ** *p* < 0.01) (Scale bars = 20 μm).

The regulatory effects of FRGE on GLUT2, a key transporter involved in luminal-to-blood glucose movement, were also assessed. FRGE reduced GLUT2 expression by approximately 1.5 times compared with RGE (Figure 3). Importantly, even in the presence of HG, GLUT2 expression in the FRGE-treated group remained comparable to that in the control group and was significantly lower than that observed in the HG condition alone. Compared with other HG-exposed groups, GLUT2 expression in FRGE-treated cells was reduced by approximately 2 times (Figure 3). Immunocytochemistry results were consistent with the flow cytometry data (Figure 3).

Based on the findings in Figure 1 and Figure 2, FRGE exhibited a significantly stronger effect compared with RGE in modulating intestinal glucose absorption, supporting its potential as an effective bioactive material for glycemic regulation.

**Figure 4:**
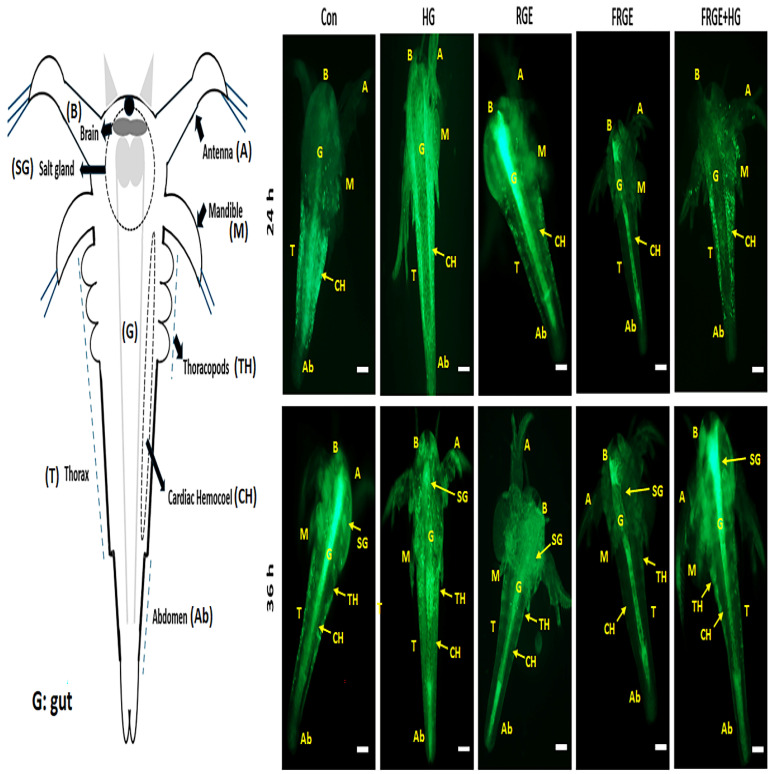
Modulation of glucose absorption by FRGE in brine shrimp. Images showing the uptake of FITC-labeled glucose by brine shrimp over time of exposure (24, 36 h), Con, control; HG, high glucose exposure; FRGE, fermented *Rehmannia glutinosa* root extract; RGE, *Rehmannia glutinosa* root extract; FRGE + HG, mixture with FRGE and HG. Dotted lines indicate anatomical regions. (scale bars = 20 μm).

The glucose absorption experiment using *Artemia* represents an effective preliminary model and serves as a suitable alternative to traditional animal experiments. In *Artemia* nauplii exposed to FRGE for 24 h, fluorescently labeled glucose was predominantly localized within the intestinal tract, whereas nauplii exposed to HG exhibited marked glucose absorption and accumulation within the cardiac hemocoel (CH) (Figure 4). After 36 h of exposure, glucose distribution showed distinct differences between groups. Nauplii treated with FRGE retained most of the fluorescent glucose within the intestine, with only weak fluorescence detected in other anatomical regions. In contrast, HG-treated nauplii absorbed substantial amounts of glucose, which accumulated across multiple organs, including the branchial chamber (B), salivary gland (SG), abdomen (A), midgut (M), and thorax (TH) (Figure 4).

### 3.2. Enhanced Immunoactivity by FRGE

**Figure 5:**
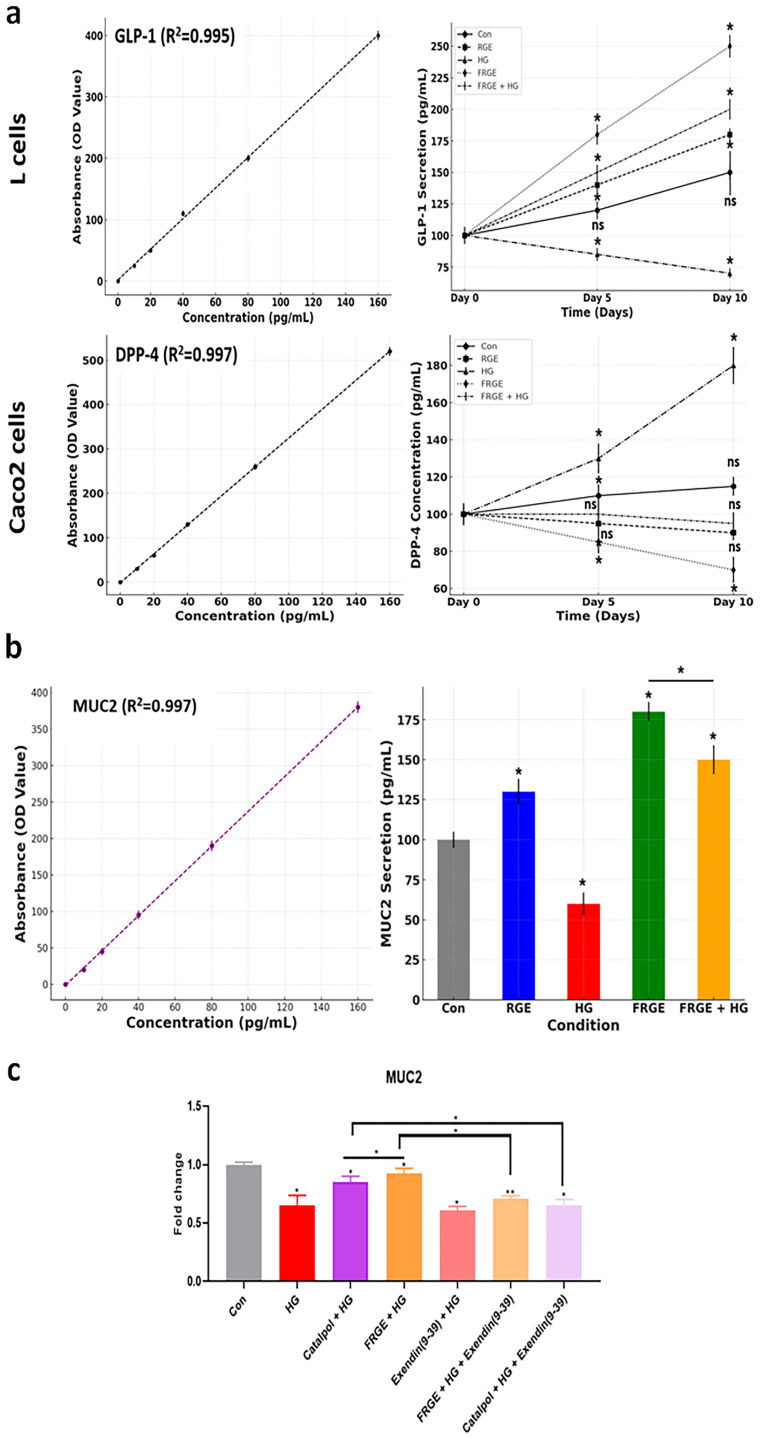
Effects of FRGE on intestinal cells involved in glucose absorption and immune modulation. (**a**) Evaluation of GLP-1 secretion from L cells and DPP-4 expression in Caco-2 intestinal cells. (**b**) Assessment of MUC2 expression in intestinal epithelial cells at day 10. (**c**) Validation of GLP-1R-dependent regulation of MUC2 expression using catalpol and a GLP-1R antagonist. Caco-2 cells were treated under the following conditions: control (Con), high glucose (HG; 25 mM), catalpol + HG, FRGE + HG, Exendin (9–39) + HG, FRGE + HG + Exendin (9–39), and catalpol + HG + Exendin (9–39), ns; not significant (* *p* < 0.05, ** *p* < 0.01).

The expression of GLP-1, a key regulator of intestinal glucose absorption and immune signaling, was evaluated. FRGE treatment resulted in a sustained increase in GLP-1 secretion over a 10-day period. Despite HG exposure, GLP-1 levels in the FRGE-treated group were approximately 1.8 times higher on day 5 and 2.9 times higher on day 10 than those in the HG group (Figure 5a). In addition, FRGE markedly suppressed the expression of DPP-4, a major enzyme responsible for GLP-1 degradation in intestinal cells. On day 10, DPP-4 levels were reduced by approximately 2.6 times compared with HG, and even under HG conditions, FRGE induced an approximately 1.8 times reduction (Figure 5a).

To examine the secretion of MUC2, an intestinal mucin that contributes to mucosal defense and is known to be stimulated by GLP-1 signaling, FRGE increased MUC2 levels by approximately 1.4 times compared with RGE and by 2.3 times under HG conditions (Figure 5b). This pattern paralleled the GLP-1 secretion results described in Figure 5a. To further validate whether the MUC2-enhancing effect of FRGE was associated with catalpol-mediated GLP-1R signaling, catalpol-only and GLP-1R inhibition experiments were performed. Under HG conditions, both catalpol and FRGE restored MUC2 expression compared with HG exposure alone, with FRGE showing a significantly greater recovery effect. However, pretreatment with Exendin (9–39), a GLP-1R antagonist, markedly attenuated the MUC2-restoring effects of both FRGE and catalpol. These results indicate that FRGE-induced MUC2 expression is at least partly mediated by catalpol-associated GLP-1R signaling (Figure 5c).

**Figure 6:**
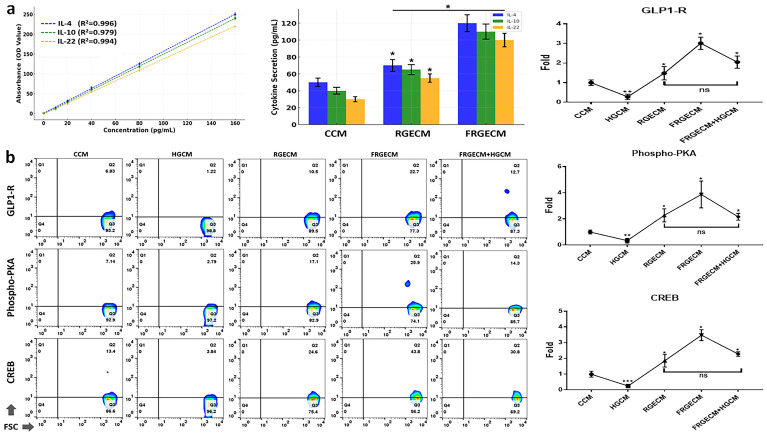
Immune modulation of macrophages under FRGE. (**a**) Evaluation of cytokine secretion (IL-4, IL-10, IL-22) in macrophages under FRGE treatment. (**b**) Expression levels of key markers associated with anti-inflammatory signaling in macrophages. CCM, control-conditioned medium; HGCM, high glucose exposure-conditioned medium; FRGECM, fermented *Rehmannia glutinosa* root extract-conditioned medium; RGECM, *Rehmannia glutinosa* root extract-conditioned medium, ns; not significant (* *p* < 0.05, ** *p* < 0.01, *** *p* < 0.001).

Macrophages exposed to conditioned media (CM) collected from FRGE-treated small intestinal cells secreted markedly higher levels of anti-inflammatory cytokines. Specifically, FRGECM increased secretion of IL-4, IL-10, and IL-22 by approximately 2.4, 1.8, and 1.7 times, respectively, compared with RGECM (Figure 6a). In parallel, intracellular signaling markers associated with inflammation regulation (GLP-1R, PKA, and CREB) were, on average, 1.7 times higher in FRGECM-treated macrophages than in RGECM-treated cells (Figure 6b). Furthermore, under HG exposure, FRGECM increased anti-inflammatory marker expression by approximately 2.7 times compared with HGCM (Figure 6b). These findings were consistent with the elevated GLP-1 secretion observed in L cells (Figure 5a).

## 4. Discussion

Fermentation is a promising biotechnological process for enhancing the pharmacological properties of natural products. It can improve the bioavailability, stability, and functional activity of phytochemicals by breaking down complex molecules into absorbable forms [1]. For instance, fermentation has been shown to increase the levels of bioactive compounds, such as polyphenols, flavonoids, and glycosides, leading to enhanced antioxidant and anti-inflammatory activities [21]. In addition, fermentation may reduce toxic compounds or allergens present in raw herbs, thereby improving their safety profiles [22]. However, the fermentation process has several limitations. It may alter or degrade some sensitive compounds, resulting in reduced therapeutic efficacy [23]. The reproducibility of fermentation outcomes may be inconsistent due to microbial strain variability, substrate composition, and process conditions [24]. Furthermore, the complexity of fermentation mechanisms poses challenges for standardizing and scaling industrial production. Therefore, careful control and validation of fermentation conditions are necessary to ensure consistent and safe outcomes. In this context, fermentation markedly enhanced the catalpol content of *R. glutinosa* extract, nearly doubling its concentration, while simultaneously reducing its iridoid precursors and increasing key metabolic enzyme activities, suggesting efficient bioconversion pathways (Figure 1). Such microbially mediated conversion of iridoid glycosides is consistent with previous findings in Lactobacillus-mediated hydrolysis and restructuring of glycoside substrates [25]. Increased secretion of GLP-1 plays a pivotal role in modulating intestinal glucose absorption and immune responses via multiple intracellular signaling pathways. One of the principal mechanisms involves the activation of GLP-1R expressed on intestinal epithelial cells, which subsequently triggers the PKA signaling cascade via cAMP accumulation [26]. Activation of PKA leads to phosphorylation of CREB, a transcription factor that regulates the expression of genes involved in glucose transport and mucosal immune responses [27]. CREB signaling may indirectly regulate intestinal glucose transport by modulating epithelial homeostasis rather than directly increasing SGLT1 and GLUT2 expression. The increase in catalpol is likely a major contributor to this effect [12]. Furthermore, PKA-CREB signaling modulates tight junction proteins, thereby influencing epithelial barrier integrity and antigen permeability [28]. In the context of immune activation, GLP-1R signaling has been shown to downregulate pro-inflammatory cytokine production, such as TNF-α and IL-6, while enhancing anti-inflammatory mediators including IL-10 [29]. These immunomodulatory effects are partly mediated by CREB-driven transcriptional activation of anti-inflammatory genes [18]. The present findings indicate significant upregulation of GLP-1R, PKA, and phosphorylated CREB in intestinal tissues, suggesting that these molecules collectively contribute to the regulation of nutrient absorption and mucosal immune homeostasis. The observed activation of the GLP-1R/PKA/CREB axis supports the hypothesis that GLP-1 not only serves as an incretin hormone but also as a modulator of gut physiology under dietary or therapeutic interventions [30]. These observations provide a molecular basis for targeting the GLP-1 pathway in metabolic and inflammatory disorders of the gastrointestinal tract. The elevation of GLP-1 observed in FRGE-treated cells aligns with the fermentation-induced catalpol enhancement, as catalpol is known to stimulate GLP-1 secretion and potentiate its downstream signaling [31]. Consistent with previous reports describing GLP-1-mediated immune regulation, the present findings suggest that fermentation-enhanced catalpol enrichment may extend these immunomodulatory effects to intestinal mucosal defense through coordinated regulation of DPP-4, MUC2, and anti-inflammatory cytokines. Although the increase in catalpol is likely a major contributor, we cannot exclude the possibility that other fermentation-derived metabolites contributed to GLP-1 signaling and cytokine modulation. The catalpol-only treatment and Exendin (9–39) inhibition results further support the involvement of catalpol-mediated GLP-1R signaling in FRGE-induced mucosal defense. GLP-1 exerts anti-inflammatory effects by promoting type 2 immune responses, including the activation of IL-4 and IL-10-producing macrophages [16]. Catalpol, an iridoid glycoside, enhances GLP-1 secretion and amplifies its downstream effects [32]. The elevation of IL-22 is particularly notable as this cytokine plays a critical role in maintaining epithelial barrier integrity and stimulating MUC2 production [19]. Consistently, we found increased MUC2 expression in the intestinal mucosa, indicating enhanced mucus barrier formation, which is essential for protection against pathogen invasion and the maintenance of gut homeostasis [20]. Furthermore, DPP-4, a major GLP-1-degrading enzyme, is downregulated in intestinal tissue, potentially contributing to the sustained activity of GLP-1 and its protective effects [33]. These results suggest that catalpol exerts its beneficial effects by potentiating GLP-1 signaling and modulating the gut immune microenvironment. The collective findings indicate that the GLP-1–catalpol axis may orchestrate both immune tolerance and mucosal protection through cytokine modulation and enhancement of epithelial defense. The precursor depletion and enzyme activation patterns demonstrated in Figure 1 further support a model in which fermentation enhances the availability of catalpol and related metabolites capable of influencing GLP-1–mediated immune pathways. Compared with previous reports on fermented *Rehmannia glutinosa*, the present work highlights several mechanistic and functional distinctions. Previous studies on fermented *Rehmannia glutinosa* mainly emphasized antioxidant activity or general anti-inflammatory effects, whereas the present study provides mechanistic evidence linking fermentation-enhanced catalpol enrichment with GLP-1R-mediated regulation of intestinal glucose transport and mucosal immune signaling. In particular, the observed modulation of SGLT1 and GLUT2 expands the currently recognized functional scope of fermented herbal extracts in metabolic regulation [34,35]. Furthermore, the present findings provide mechanistic insight into glucose absorption regulation and immune activation. Specifically, the fermented extract suppressed SGLT1 and GLUT2 expression in intestinal epithelial cells, thereby reducing glucose uptake, a mechanism not addressed in earlier studies. This study is the first to report that FRGE enhances GLP-1 secretion and suppresses DPP-4 expression, thereby extending the bioactivity of this critical incretin hormone. Notably, FRGECM stimulated macrophage-derived anti-inflammatory cytokines (IL-4, IL-10, and IL-22) and activated downstream GLP-1R/PKA/CREB signaling pathways, which have not been previously explored in the context of fermented herbal extracts. Furthermore, the use of *A. nauplii* as an alternative in vivo model offers a novel and ethical approach for visualizing glucose absorption. These findings suggest that FRGE possesses dual functionality in metabolic regulation and immune modulation and establishes its potential as a functional food ingredient with broad therapeutic applications. The fermentation-driven enhancement of catalpol content and precursor conversion observed in Figure 1 provides a biochemical foundation that helps explain the superior biological activities of FRGE compared with RGE. These cellular findings were further supported by in vivo visualization using *Artemia* nauplii, in which FRGE markedly reduced systemic glucose accumulation, consistent with its inhibitory effects on intestinal glucose transport. Although the Artemia model does not fully replicate mammalian intestinal physiology, it provides a rapid, cost-effective, and ethically favorable alternative animal-free experimental platform for preliminary visualization of glucose absorption dynamics. Nevertheless, further validation using mammalian models will be necessary to confirm the translational relevance of FRGE-mediated metabolic regulation.

## 5. Conclusions

This study provides evidence that FRGE exerts dual functionality by modulating both metabolic and immune pathways. FRGE reduced intestinal glucose absorption by downregulating SGLT1 and GLUT2, while simultaneously enhancing GLP-1 secretion and suppressing its degradation by DPP-4. These effects were consistent with the fermentation-induced increase in catalpol and the elevated activities of enzymes involved in iridoid bioconversion. This activated the GLP-1R/PKA/CREB signaling axis, leading to increased MUC2 expression and higher secretion of macrophage-derived anti-inflammatory cytokines, including IL-4, IL-10, and IL-22. Together, these findings highlight a coordinated mechanism whereby fermentation enhances catalpol availability, potentiates GLP-1 signaling, and strengthens both metabolic regulation and mucosal immune responses. Thus, FRGE emerges as a promising bioactive material with potential applications in functional foods targeting metabolic and gastrointestinal health. Despite these strengths, this study has limitations, including variability in fermentation processes, the need for broader validation using additional GLP-1R downstream inhibitors or DPP-4-modulating controls, and the focus on macrophages without evaluating other immune cell types. Industrially, these results underscore the potential of fermentation as a value-adding biotechnological strategy to enhance the functional properties of *R. glutinosa*. By improving its bioactivity and safety, FRGE may serve as a novel ingredient in nutraceuticals for metabolic syndrome, type 2 diabetes, and inflammatory bowel conditions. Future studies should validate catalpol’s specific contribution using purified-compound controls, characterize additional fermentation-derived metabolites, and standardize fermentation processes to support clinical translation and commercial application. These findings provide a scientific foundation for the development of FRGE as a functional food ingredient targeting metabolic and gastrointestinal health. However, several translational limitations remain, including variability in fermentation-derived metabolite composition, uncertainty regarding oral bioavailability, and the need for dose standardization and long-term safety evaluation. Therefore, additional preclinical studies using mammalian models will be necessary before clinical application of FRGE can be considered.

## Figures and Tables

**Table 1:** Quantitative analysis of catalpol in *Rehmannia glutinosa* extracts fermented with different microbial strains.

Strain	Pre-Fermentation Catalpol (µg/mL)	Post-Fermentation Catalpol (µg/mL)
*Saccharomyces cerevisiae* ATCC 13007	60.0 ± 3.0 a	85.0 ± 5.0 b *
*L. plantarum* ATCC 8014 (LP1)	62.0 ± 4.0 a	91.4 ± 6.0 b *
*L. plantarum* KCCM 43246 (LP2)	62.4 ± 3.5 a	116.7 ± 4.0 c **

Different lowercase letters indicate significant differences among post-fermentation groups (*p* < 0.05). Asterisks indicate significant differences compared with pre-fermentation values within the same strain (* *p* < 0.05, ** *p* < 0.01).

## Data Availability

The data presented in this study are available from the corresponding author upon reasonable request.

## Abbreviations

The following abbreviations are used in this manuscript:

FRGE	Fermented *Rehmannia glutinosa* extract
RGE	*Rehmannia glutinosa* extract
GLP-1	Glucagon-like peptide-1
GLP-1R	Glucagon-like peptide-1 receptor
DPP-4	Dipeptidyl peptidase-4
MUC2	Mucin 2
SGLT1	Sodium–glucose cotransporter 1
GLUT2	Glucose transporter 2
PKA	Protein kinase A
CREB	cAMP response element-binding protein
HG	High glucose
Con	Control
CM	Conditioned medium
FRGECM	Fermented *Rehmannia glutinosa* extract-conditioned medium
RGECM	*Rehmannia glutinosa* extract-conditioned medium
HGCM	High glucose-conditioned medium
IL	Interleukin
GF	General fermentation
SF	Specific fermentation

## References

[1] Hussain A., Bose S., Wang J.-H., Yadav M.K., Mahajan G.B., Kim H. (2016). Fermentation, a feasible strategy for enhancing bioactivity of herbal medicines. Food Res. Int..

[2] Yang H.-Y., Han L., Lin Y.-Q., Li T., Wei Y., Zhao L.-H., Tong X.-L. (2023). Probiotic fermentation of herbal medicine: Progress, challenges, and opportunities. Am. J. Chin. Med..

[3] Bai Y., Zhu R., Tian Y., Li R., Chen B., Zhang H., Xia B., Zhao D., Mo F., Zhang D. (2019). Catalpol in diabetes and its complications: A review of pharmacology, pharmacokinetics, and safety. Molecules.

[4] Yan J., Wang C., Jin Y., Meng Q., Liu Q., Liu Z., Liu K., Sun H. (2018). Catalpol ameliorates hepatic insulin resistance in type 2 diabetes through acting on AMPK/NOX4/PI3K/AKT pathway. Pharmacol. Res..

[5] Cao Q., Wang Z., Jiang Y., Dong C. (2024). *Rehmannia glutinosa* polysaccharides: A review on structure–activity relationship and biological activity. Med. Chem. Res..

[6] Yang C., Shi Z., You L., Du Y., Ni J., Yan D. (2020). Neuroprotective effect of catalpol via anti-oxidative, anti-inflammatory, and anti-apoptotic mechanisms. Front. Pharmacol..

[7] Oudin A., Courtois M., Rideau M., Clastre M. (2007). The iridoid pathway in *Catharanthus roseus* alkaloid biosynthesis. Phytochem. Rev..

[8] Amer A., Kashfa B., Bibi A. (2017). Microbial β-glucosidase: Sources, production and applications. J. Appl. Environ. Microbiol..

[9] Martínez A.T., Ruiz-Dueñas F.J., Camarero S., Serrano A., Linde D., Lund H., Vind J., Tovborg M., Herold-Majumdar O.M., Hofrichter M. (2017). Oxidoreductases on their way to industrial biotransformations. Biotechnol. Adv..

[10] Switzer A. (2018). Microbial beta-glucosidases and their roles in glycoside hydrolysis. Crit. Rev. Biotechnol..

[11] Mazzoli R. (2014). Microbial esterases and oxidoreductases involved in biotransformation pathways. Appl. Microbiol. Biotechnol..

[12] Röder P.V., Geillinger K.E., Zietek T.S., Thorens B., Koepsell H., Daniel H. (2014). The role of SGLT1 and GLUT2 in intestinal glucose transport and sensing. PLoS ONE.

[13] Zheng Z., Zong Y., Ma Y., Tian Y., Pang Y., Zhang C., Gao J. (2024). Glucagon-like peptide-1 receptor: Mechanisms and advances in therapy. Signal Transduct. Target. Ther..

[14] Drucker D.J. (2018). Mechanisms of action and therapeutic application of glucagon-like peptide-1. Cell Metab..

[15] Lee Y.-S., Park M.-S., Choung J.-S., Kim S.-S., Oh H.-H., Choi C.-S., Ha S.-Y., Kang Y., Kim Y., Jun H.-S. (2012). Glucagon-like peptide-1 inhibits adipose tissue macrophage infiltration and inflammation in an obese mouse model of diabetes. Diabetologia.

[16] Chen J., Mei A., Wei Y., Li C., Qian H., Min X., Yang H., Dong L., Rao X., Zhong J. (2022). GLP-1 receptor agonist as a modulator of innate immunity. Front. Immunol..

[17] Shin H., Kim H., Kim G., Kim Y., Kim B. (2025). Enhanced Bioactivity of Fermented Aralia cordata Extract for Glucose and Immune Modulation. Curr. Issues Mol. Biol..

[18] Sanin D.E., Prendergast C.T., Mountford A.P. (2015). IL-10 production in macrophages is regulated by a TLR-driven CREB-mediated mechanism that is linked to genes involved in cell metabolism. J. Immunol..

[19] Aujla S.J., Chan Y.R., Zheng M., Fei M., Askew D.J., Pociask D.A., Reinhart T.A., McAllister F., Edeal J., Gaus K. (2008). IL-22 mediates mucosal host defense against Gram-negative bacterial pneumonia. Nat. Med..

[20] Pelaseyed T., Bergström J.H., Gustafsson J.K., Ermund A., Birchenough G.M., Schütte A., van der Post S., Svensson F., Rodríguez-Piñeiro A.M., Nyström E.E. (2014). The mucus and mucins of the goblet cells and enterocytes provide the first defense line of the gastrointestinal tract and interact with the immune system. Immunol. Rev..

[21] Oh Y.-C., Cho W.-K., Jeong Y.H., Im G.Y., Yang M.C., Ma J.Y. (2012). Fermentation improves anti-inflammatory effect of sipjeondaebotang on LPS-stimulated RAW 264.7 cells. Am. J. Chin. Med..

[22] Joo S.S., Won T.J., Nam S.Y., Kim Y.B., Lee Y.C., Park S.Y., Park H.Y., Hwang K.W., Lee D.I. (2009). Therapeutic advantages of medicinal herbs fermented with *Lactobacillus plantarum*, in topical application and its activities on atopic dermatitis. Phytother. Res. Int. J. Devoted Pharmacol. Toxicol. Eval. Nat. Prod. Deriv..

[23] Mosele J.I., Macià A., Romero M.-P., Motilva M.-J. (2016). Stability and metabolism of Arbutus unedo bioactive compounds (phenolics and antioxidants) under in vitro digestion and colonic fermentation. Food Chem..

[24] Benkerroum N. (2013). Traditional fermented foods of North African countries: Technology and food safety challenges with regard to microbiological risks. Compr. Rev. Food Sci. Food Saf..

[25] Park C.-M., Kim G.-M., Cha G.-S. (2021). Biotransformation of flavonoids by newly isolated and characterized Lactobacillus pentosus NGI01 strain from kimchi. Microorganisms.

[26] Drucker D.J. (2006). The biology of incretin hormones. Cell Metab..

[27] Mayr B., Montminy M. (2001). Transcriptional regulation by the phosphorylation-dependent factor CREB. Nat. Rev. Mol. Cell Biol..

[28] Suzuki T. (2013). Regulation of intestinal epithelial permeability by tight junctions. Cell. Mol. Life Sci..

[29] Hogan A.E., Tobin A., Ahern T., Corrigan M., Gaoatswe G., Jackson R., O’reilly V., Lynch L., Doherty D., Moynagh P.N. (2011). Glucagon-like peptide-1 (GLP-1) and the regulation of human invariant natural killer T cells: Lessons from obesity, diabetes and psoriasis. Diabetologia.

[30] Liu Q.K. (2024). Mechanisms of action and therapeutic applications of GLP-1 and dual GIP/GLP-1 receptor agonists. Front. Endocrinol..

[31] Zhang B., Weston L.A., Li M., Zhu X., Weston P.A., Feng F., Zhang B., Zhang L., Gu L., Zhang Z. (2020). *Rehmannia glutinosa* replant issues: Root exudate-rhizobiome interactions clearly influence replant success. Front. Microbiol..

[32] Habtemariam S. (2017). Antidiabetic potential of monoterpenes: A case of small molecules punching above their weight. Int. J. Mol. Sci..

[33] Mulvihill E.E., Drucker D.J. (2014). Pharmacology, physiology, and mechanisms of action of dipeptidyl peptidase-4 inhibitors. Endocr. Rev..

[34] Ren H., Li K., Min Y., Qiu B., Huang X., Luo J., Qi L., Kang M., Xia P., Qiao H. (2023). *Rehmannia glutinosa* polysaccharides: Optimization of the decolorization process and antioxidant and anti-inflammatory effects in LPS-stimulated porcine intestinal epithelial cells. Antioxidants.

[35] Kim E.-H., Kim K.-S., Chae S.-K., Kim B.-S., Kang J.-S. (2012). Comparison of biological activities on Rehmanniae radix and fermented Rehmanniae radix. J. Physiol. Pathol. Korean Med..

